# Taux des plaquettes sanguines en phase intercritique et expressivité clinique de la drépanocytose dans un centre de référence de la drépanocytose au Mali

**DOI:** 10.11604/pamj.2022.43.52.32674

**Published:** 2022-10-03

**Authors:** Lamine Diallo, Aldiouma Guindo, Ibrahima Kéita, Mohamed Ag Baraïka, Abdoul Karim Dembélé, Boubacari Ali Touré, Dapa Aly Diallo

**Affiliations:** 1 Centre de Recherche et de Lutte contre la Drépanocytose, Bamako, Mali

**Keywords:** Complications drépanocytaires, facteurs de risque, plaquettes sanguines, Complications of sickle cell disease, risk factors, platelets

## Abstract

Les facteurs de risque associés aux complications drépanocytaires sont insuffisamment élucidés. L´objectif de notre étude était de vérifier l´existence d´une association entre l´expressivité clinique de la drépanocytose et le taux des plaquettes sanguines en phase intercritique chez des drépanocytaires en suivi au Centre de Recherche et de Lutte contre la Drépanocytose de Bamako au Mali. Quarante dossiers de patients drépanocytaires âgés de 5 à 42 ans du Centre de Recherche et de Lutte contre la Drépanocytose de Bamako au Mali ont fait rétrospectivement, l´objet de l´étude. L´expressivité des cas a été appréciée selon les critères de CVO et/ou d´hospitalisations < ou ≥ 2 par an. La saisie des données a été réalisée à l´aide du logiciel Excel dans sa version de 2013. Les tests statistiques utilisés ont été les tests Chi2, de Student et de Mac Nemar. Parmi les 40 patients, 82,5% étaient du phénotype hémolytique et 17,5%, du phénotype hypervisqueux; les complications drépanocytaires étaient plus fréquentes dans le groupe du phénotype hémolytique (p < 0,05). Il existait une association significative entre un taux moyen des plaquettes ≥ 450 G/L en phase intercritique et un nombre annuel de CVO ≥ 2 (p = 0,002). Ce travail montre qu´un taux moyen de plaquettes sanguines ≥ 450 G/L chez le drépanocytaire en phase intercritique serait un facteur de risque de survenue fréquente de CVO. Il souligne l´intérêt de conduire d´une part, des études prospectives, considérant à la fois le paramètre hyperplaquettose sanguine et marqueurs de l´activation plaquettaire sur des échantillons de taille plus importante, d´autre part, des essais thérapeutiques impliquant les inhibiteurs de l´activation plaquettaire comme le Crizanlizumab, un anticorps monoclonal humanisé anti-P-sélectine.

## Abstract (en)

Risk factors associated with complications occurring in sickle cell disease are not fully elucidated. The purpose of this study was to evaluate the existence of an association between the clinical severity of sickle cell disease and platelet count in the steady state phase in patients with sickle cell disease followed up at the Center for Research and Control of Sickle Cell Disease in Bamako, Mali. We conducted a retrospective review of 40 medical records of patients aged 5 to 42 years with sickle cell disease at the Center for Research and Control of Sickle Cell Disease in Bamako, Mali. Clinical severity of sickle cell disease was assessed according to the criteria of VOC and/or hospitalizations < 2 or ≥ 2 per year. Data entry was carried out using the Excel 2013 version. The statistical tests used were the Chi2, Student and Mac Nemar tests. Of the 40 patients, 82.5% had haemolytic phenotype and 17.5% hyperviscous phenotype; complications of sickle cell disease were more frequent in the haemolytic phenotype group (p < 0.05). There was a significant association between mean platelet count ≥ 450 G/L in the steady state phase and the annual number of CVOs ≥ 2 (p = 0.002). This study shows that mean platelet count ≥ 450 G/L in sickle cell patients in the steady state phase could be a risk factor for the frequent occurrence of CVO. It underlines the importance of conducting prospective studies focusing on both hyperplateletosis and platelet activation markers in larger sample sizes, as well as therapeutic trials involving platelet activation inhibitors, such as Crizanlizumab, a humanised anti-P-selectin monoclonal antibodies.

## Introduction

La drépanocytose figure parmi les hémoglobinopathies les plus répandues dans le monde. Chaque année, plus de 500 000 nouveau-nés naissent dans le monde avec un syndrome drépanocytaire majeur [1,2]. L´hémoglobine S anormale qui la caractérise, sous certaines conditions notamment en cas de baisse de la pression partielle du sang en oxygène, polymérise et forme de longues fibres rigides responsables d´une déformation du globule rouge en faucille (falciformation) dont la déformabilité est diminuée. La falciformation des globules rouges et la réduction de leur déformabilité induisent une obstruction au niveau de la microcirculation capillaire et des infarctus d´organes responsables de crises douloureuses dont la manifestation la plus fréquente est la crise vasoocclusive (CVO) simple [3]. Parfois la crise se manifeste sous forme de complications aiguës à type de syndrome thoracique aigue (STA), d´accident vasculaire cérébral (AVC), d´hématurie, etc. [4-6]. La répétition des phénomènes obstructifs de la microcirculation capillaire aboutit à des complications chroniques, responsables de défaillances d´organes. Les facteurs de risque associés à ces complications ne sont pas encore entièrement élucidés. En fonction de certaines complications, on a pu catégoriser les phénotypes d´expressivité clinique en phénotype hémolytique regroupant les drépanocytaires SS et S/β^0^thalassémiques et hypervisqueux, regroupant les drépanocytaires SC et S/β^+^thalassémiques [7,8]. Il a été rapporté qu´au cours des CVO, toutes les composantes cellulaires notamment les globules blancs et les plaquettes étaient activées et quelques études ont rapporté que le volume plaquettaire moyen (VPM) marqueur fiable de la fonction plaquettaire, est un facteur associé aux CVO et aux infarctus cérébraux [9-11]. A notre connaissance, aucune étude ne documente une telle association en Afrique au sud du Sahara. L´objectif de l´étude était de vérifier l´existence d´une association entre l´expressivité clinique de la drépanocytose et le taux des plaquettes sanguines en phase intercritique chez des drépanocytaires en suivi au Centre de Recherche et de Lutte contre la Drépanocytose de Bamako au Mali.

## Méthodes

L´étude est une rétrospective ayant porté sur les dossiers de patients drépanocytaires âgés de 5 ans et au-delà, sans distinction de genre et suivis en cohorte de 2016 à 2018, au Centre de Recherche et de Lutte contre la Drépanocytose (CRLD) de Bamako au Mali. La sélection des dossiers était consécutive sur la base de critères préétablis: dossier de patient drépanocytaire quel que soit son génotype drépanocytaire; dossier de patient ayant fait au moins une crise drépanocytaire par an; dossier de patient inscrit au CRLD au cours de la période d´étude, dossier de patient ayant fait au moins une consultation de suivi par an au CRLD. Ainsi 40 dossiers de patients drépanocytaires ont été retenus pour être analysés. N´étaient pas inclus les dossiers des patients ne répondant pas aux critères d´inclusion ci-dessus, sous traitement anticoagulant ou antiagrégant, ou traités par Hydroxyurée (Hydrea®). Un formulaire de rapport des cas anonymisé a permis de recueillir les paramètres sociodémographiques des participants à l´étude (le sexe, l´âge, l´ethnie), les génotypes drépanocytaires des patients. Ont été considérés, les hémogrammes faits au moment des crises drépanocytaires et en phase intercritique. Les automates de numération globulaire étaient ABX Micros 60 et le Yumizen H500. La crise aigüe simple ou compliquée était documentée selon les procédures du Centre à partir de l´examen clinique lors de la consultation et des examens complémentaires comprenant outre des examens biologiques, des examens d´imagerie médicale (radiographies du thorax et du bassin, l´échographie abdominale et ou pelvienne et le doppler transcrânien). La saisie des données a été réalisée à l´aide du logiciel REDcap version 10.3.3. Les analyses statistiques ont été réalisées à l´aide du logiciel R studio version 3.5.2. Les variables qualitatives ont été présentées en pourcentage et les variables quantitatives en moyenne ± écart-type. Les associations entre le taux moyen des plaquettes chez le patient en phase intercritique et le nombre annuel de CVO ou d´hospitalisations ont été étudiées en prenant le patient comme son propre témoin. Les tests de Chi2, de Student et de Mac Nemar ont été utilisés dans les conditions appropriées. Pour la comparaison des moyennes et des fréquences, une valeur de p inférieure à 0,05 était considérée comme statistiquement significative.

**Considérations éthiques:** l´étude a obtenu l´approbation du Comité d´éthique institutionnel (CEI) du Centre d Recherche et de Lutte contre la Drépanocytose de Bamako.

**Financement:** ce travail a été financé sur les fonds de recherche du CRLD et grâce à l´appui financier de la Fondation Pierre Fabre ayant permis la saisie des données sur le logiciel REDcap.

## Résultats

La cohorte étudiée comptait 24 femmes et 16 hommes dont l´âge était compris entre 5 et 42 ans. La tranche d´âge de 5 à 10 ans (23/40: 75,5%) était plus représentée. Parmi les génotypes drépanocytaires on comptait 23 SS (70%), 5 S/β^0^thalassémiques (12,5%), 6 SC (15%) et 1 S/β+thalassémique (2,5%). Soit 82,5% de phénotypes cliniques hémolytiques (SS et S/ β^0^thalassémiques) et 17,5% de phénotypes cliniques hypervisqueux (SC et S/β+thalassémiques). Le nombre de consultations systématiques de suivi par patient était compris entre 1 et 4; vingt-un patients (52,5%) avaient fait une consultation de suivi dans l´année, 47,5% au moins deux consultations. Les complications enregistrées étaient dominées par les CVO simples avec une fréquence annuelle au moins égale à 2 chez 67,5% (27/40) et à 3 chez 35% (14/40) des patients. De façon générale les génotypes SS et S β^0^ thalassémiques étaient les plus expressifs. Le nombre d´hospitalisations annuel variait entre 0 et 7. Un patient sur deux avait été hospitalisé au moins 2 fois dans l´année et 1 patient sur 3 l´avait été au moins 3 fois dans l´année. La médiane de fréquence des hospitalisations était de 5. Le taux moyen des plaquettes était significativement plus bas au moment des crises aiguës qu´en phase intercritique (401,13 ± 138 G/L contre 456,15 ± 164 G/L: p = 0,02). Un taux de plaquettes ≥ 450 G/L était significativement plus fréquente en phase intercritique (p = 0,001) qu´au cours des CVO (Tableau 1). L´étude des associations entre le taux des plaquettes chez le patient en phase intercritique et le nombre annuel de CVO ou d´hospitalisations en prenant le patient comme son propre témoin, montrait qu´un taux de plaquettes ≥ 450 G/L en phase intercritique, était positivement corrélé au nombre annuel de CVO mais inversement corrélé au nombre d´hospitalisations ≥ 2/an. Dans les mêmes conditions, un VPM > 7 FL était significativement associé à un nombre annuel de CVO et d´hospitalisations ≥ 2 (Tableau 2).

**Tableau 1:** fréquences comparées du taux des plaquettes ≥ 450 G/L en période de CVO et en phase intercritique

	CVO		
Plaquettes ≥ 450 G/L	Non	Oui	p
Non	16	0	0,0015
Oui	12	12	
Total	28	12	40

**Tableau 2:** associations entre le taux des plaquettes ≥ 450 G/L ou un VPM > 7 FL chez le patient en phase intercritique et le nombre annuel de CVO ou d’hospitalisations (patient pris comme son propre témoin)

Paramètres	Nombre de CVO/an			Nombre d'hospitalisations/an		
	≥ 2	< 2	p	≥ 2	< 2	p
Plaquettes ≥ 450 G/L	17	7	0,01419	11	13	0,00738
Plaquettes < 450 G/L	10	6	0,3074	9	7	0,1747
Total	27	13	-	20	20	-
VPM ≤ 7 FL	10	3	0,09609	6	7	0,5672
VPM > 7 FL	17	10	0,002032	14	13	0,001091
Total	27	13	-	20	20	-

## Discussion

Ce travail a porté sur une population de drépanocytaires jeunes ayant un phénotype clinique plus souvent hémolytique qu´hypervisqueux. Cette distribution reflète celle du Centre de recrutement et confirme celle observée ailleurs en Afrique au sud du Sahara, du moins dans les pays loin du plateau voltaïque [12-16]. Il a permis de constater que les complications drépanocytaires chez les patients étudiés étaient dominées par les CVO. La majorité des patients soit 67,5%, avaient fait au moins 2 CVO/an alors que 35% en avaient fait au moins 3 avec des extrêmes de 1 et plus de 8. Le nombre annuel d´hospitalisations a varié entre 1 et 7 selon les patients; 50% avaient été hospitalisés au moins 2 fois dans l´année. Il s´agit donc globalement d´une population de drépanocytaires expressifs. La comparaison des phénotypes drépanocytaires montre que les phénotypes hémolytiques (SS/ Sβ^0^ thalassémie) étaient plus expressifs que les phénotypes hypervisqueux (SC/Sβ^+^ thalassémie); lorsqu´on considérait un nombre annuel moyen de CVO supérieur à 3, on observait 42,4% de patients porteurs du phénotype hémolytique contre 0% des patients du phénotype hypervisqueux (p < 0,001). Les patients de phénotype hémolytique faisaient plus souvent des CVO ≥ 2 /an (p = 0,03) ou d´hospitalisations à une fréquence ≥ 2 /an (p = 0,02) que ceux du phénotype hypervisqueux. Ces constats sont ceux rapportés dans la littérature en général [17-20]. Nous avons observé un taux de plaquettes sanguines ≥ 450 G/L chez 60% des patients inclus dans l´étude, un constat similaire a été fait par Dahmani *et al*. au Maroc en 2016 à propos d´une cohorte de 87 drépanocytaires [21]. Des auteurs ghanéens ont rapporté un taux de plaquettes significativement plus élevé chez le drépanocytaire en crise ou non, comparés à des sujets non drépanocytaires [22]. Les taux moyens des plaquettes étaient significativement plus élevés en phases intercritiques (p = 0,01) qu´au cours des CVO et il était apparu également qu´un taux de plaquettes ≥ 450 G/L était significativement associé à la phase intercritique (p < 0,001). Ce constat est nouveau à notre connaissance. Certains auteurs ont rapporté un état d´hypercoagulabilité chez les drépanocytaires impliquant une activation plaquettaire qui augmente dramatiquement en cas de CVO avec libération d´E-sélective, de la P-sélective, de l´ICAM-1 soluble et du facteur tissulaire [23-27]. On peut penser qu´une hypercoagulabilité plus importe au cours de la CVO responsable de l´activation des plaquettes et donc, de leur consommation expliquerait le constat; nous n´avons pas au cours de cette étude, analysé les marqueurs d´activation des plaquettes tels que l´E-sélectine, la P-sélectine, l´antigène CD63, les chimiokines PF4 et RANTES. Pour soutenir cette hypothèse, nous nous sommes intéressés au VPM décrit comme un marqueur de l´activation plaquettaire et avons constaté qu´une valeur de ce paramètre > 7FL en phase intercritique, était significativement associée à un nombre annuel de CVO ou d´hospitalisations ≥ 2. Au cours de cette étude, nous avons voulu savoir s´il y avait un risque de survenue plus fréquente de CVO ou d´hospitalisations chez les patients qui avaient un taux des plaquettes élevé (≥ 450G/L) en phase intercritique. Nous avons dans cette démarche, étudié les associations en considérant le patient comme son propre témoin. Le test de Mac Nemar nous a permis de constater qu´il existait une association significative positive entre un taux des plaquettes ≥ 450G/L et un nombre annuel de CVO ≥ 2 (p = 0,01), mais négative avec un nombre annuel d´hospitalisations ≥ 2 (p = 0,005). On peut donc conclure qu´un taux moyen de plaquettes sanguines ≥ 450 G/L chez un drépanocytaire en phase intercritique est un facteur de risque de survenue fréquente de CVO mais pas d´hospitalisations. Ces résultats suggèrent l´implication des plaquettes sanguines dans les mécanismes de survenue de la CVO et soulignent l´intérêt de conduire des essais thérapeutiques impliquant les inhibiteurs de l´activation plaquettaire en particulier le Crizanlizumab, un anticorps monoclonal humanisé anti-P-sélectine [28].

Limitations: à notre connaissance, cette étude est la première qui analyse l´association des variations du taux de plaquettes avec le nombre annuel de CVO ou d´hospitalisations en considérant le malade comme son propre témoin. Il nous semble devoir souligner toute fois que ce travail connait quelques insuffisances: d´une part, il s´agit d´une étude rétrospective, d´autre part, il n´analyse pas chez la population des drépanocytaires étudiée, les autres facteurs qui modulent l´expressivité clinique de la drépanocytose [29]. Ces insuffisances peuvent constituer un facteur limitant dans l´interprétation des résultats obtenus lorsqu´on aborde la question générale des facteurs de risque de survenue d´une CVO drépanocytaire ou d´une hospitalisation du patient drépanocytaire.

## Conclusion

Cette étude consacrée à évaluer l´expressivité clinique en fonction d´un seul de plaquettes ≥ 450G/L dans le sang chez le drépanocytaire en phase intercritique, a concerné 40 drépanocytaires en suivi au CRLD de Bamako au Mali. Elle a permis de constater une association significative entre ce paramètre en phase intercritique chez le drépanocytaire et une fréquence de CVO ≥ 2/an. Nous concluons qu´un taux de plaquettes sanguines ≥ 450G/L chez le drépanocytaire en phase intercritique est un facteur de risque de survenue fréquente de CVO chez le patient drépanocytaire. Ces résultats incitent à conduire d´autres études dans une approche prospective, considérant à la fois le taux des plaquettes dans le sang et les marqueurs de l´activation plaquettaire sur des échantillons de plus grande taille. Ils soulignent également l´intérêt de conduire des essais thérapeutiques impliquant les inhibiteurs de l´activation plaquettaire en particulier le Crizanlizumab, un anticorps monoclonal humanisé anti-P-sélectine pour optimiser les résultats de la prise en charge des patients drépanocytaires en Afrique.

### Etat des connaissances sur le sujet

- Il a été rapporté qu´au cours des CVO, toutes les composantes cellulaires notamment les globules blancs et les plaquettes étaient activées;
- Quelques études ont rapporté que le volume plaquettaire moyen (VPM) marqueur fiable de la fonction plaquettaire, est un facteur associé aux CVO et aux infarctus cérébraux;
- Le rôle de la variation du taux des plaquettes dans l´expressivité clinique de la drépanocytose n´a pas été suffisamment élucidé, notamment en Afrique subsaharienne.

### Contribution de notre étude à la connaissance

- Ce travail est le premier en Afrique subsaharienne qui montre qu´un taux moyen de plaquettes ≥ 450 G/L chez le drépanocytaire en phase intercritique peut être un facteur de risque de survenue fréquente de CVO;
- Le travail souligne l´intérêt de conduire d´une part, des études prospectives considérant à la fois le paramètre hyperplaquettose sanguine et marqueurs de l´activation plaquettaire sur des échantillons de taille plus importante, d´autre part, des essais thérapeutiques impliquant les inhibiteurs de l´activation plaquettaire comme le Crizanlizumab, un anticorps monoclonal humanisé anti-P-sélectine pour optimiser les résultats de la prise en charge des drépanocytaires en Afrique.

## Remerciements

Les auteurs remercient tout le personnel médical chargé du suivi clinique des drépanocytaires et de laboratoire de biologie du Centre de Recherche et de Lutte contre la Drépanocytose (CRLD). Les auteurs remercient la Fondation Pierre Fabre pour son appui financier à la création et au fonctionnement d´une cellule de biostatistique au CRLD qui a assuré la saisie des données sur REDcap.
